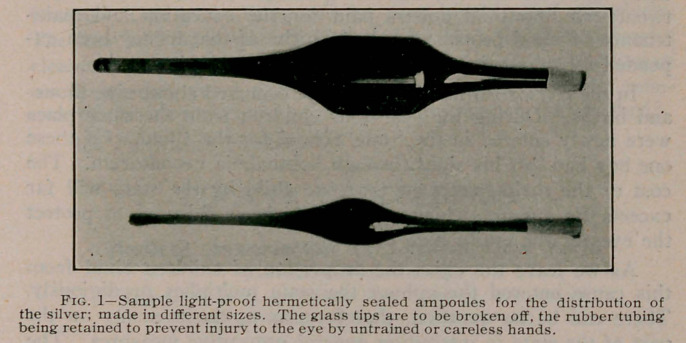# A Practical Method of Abolishing the Cause of One-Quarter of the Unnecessary Blindness in the United States

**Published:** 1906-06

**Authors:** F. Park Lewis

**Affiliations:** Buffalo, N. Y.; President New York State Commission for Improving the Condition of the Blind, 1903-1904. 454 Franklin Street


### SPECIAL SELECTION.

### A Practical Method of Abolishing the Cause of One-Quarter of the Unnecessary Blindness in the United States.

By F. PARK LEWIS, M.D., Buffalo, N. Y.

President New York State Commission for Improving the Condition of the Blind, 1903-1904.

[*Journal American Medical Association, April 28, 1906.*]

WHEN an enlightened, civilised and progressive nation quietly and passively, year after year, permits a multitude of its people unnecessarily to become blind, and more especially when one-quarter of these are infants, the reason for such a startling condition of affairs demands explanation. That such is the fact practically all reliable ophthalmologists agree.

From a summary of carefully tabulated statistics it has been demonstrated that at least four-tenths of all existing blindness might have been avoided had proper preventative or curative measures been employed, while one-quarter of this, or one-tenth of the whole, is due to ophthalmia neonatorum, an infectious, preventable and almost absolutely curable disease. Perhaps this statement will take on a new meaning when it is added that there are in the State of New York alone more than six thousand and in the United States more than fifty thousand blind people; of these, six hundred in the one state and five thousand in the country would have been saved from lives of darkness and unhappiness, in having lost all the joys that come through sight, and of more or less complete dependence, for no individual can be as self-sufficient without as with eyes—if a simple, safe and easily applied precautionary measure had been taken at the right time and in the right way to prevent this affliction. The following three vital facts are not questioned, but are universally accepted by those qualified to know:

1. The ophthalmia of infancy is an infectious germ disease.
2. By the instillation of a silver salt in the eyes of a new-born infant the disease is prevented from developing in all but an exceedingly small number of the cases in which it would otherwise have appeared.

3. In practically all those few exceptional cases the disease is absolutely curable if like treatment is employed at a sufficiently early period.

Since these facts are no longer subjects of discussion, but are universally accepted by all educated medical men, the natural inquiry follows: Why, as a commonsense proposition, are not these simple, harmless, preventive measures invariably employed, and why, in consequence of this neglect, does a nation sit quietly and indifferently by, making no attempt to prevent this enormous and needless waste of human eyes?

The reasons are threefold and lie, first, with the medical profession; second, with the lay public; third, with the state.

The medical profession, great as have been its advances during recent years and strenuous as have been the efforts of its teachers and leaders to promulgate the fundamental importance of germs in disease, is by no means yet universally familiar with the facts concerning infantile ophthalmia, as to its prevalence, its dangers, its prevention and measures that may be successfully instituted for its treatment. While the total number of cases is large, the disease may occur very rarely in the experience of any individual physician, even though he may have had an extensive general practice. When it does occur, unless the physician is fully informed, he does not anticipate it and is unprepared to meet it. He thinks in many cases, if his attention is called to the baby's eyes, as, indeed it may not be at all, that the redness and watering is caused by a trifling catarrhal conjunctivitis, and prescribes some simple collyrium or external wash for the lids. He may not see the child again for a week, when perhaps the disease is fully developed, the cornea broken down and irreparable damage done; or, as sometimes happens, he does not know of the special value of the silver salts or fears to employ them because of their possible danger to the delicate eye of the child, and prevention is omitted and correct treatment neglected. It has been shown that the larger proportion of cases of blindness resulting from infantile ophthalmia occurs in the more remote country districts where the parturient patient is infrequently seen and where preventive measures are most imperative. It may not seem possible to the progressive up-to-date practitioner that many physicians are not familiar with this common disease, but the large number of cases of infantile ophthalmia that are constantly occurring, with the clinical histories accompanying them, together with immense number of blind eyes as a direct sequence, prove beyond question that this is a fact. ternity has for her, and only when the truth is told to her that the lay public. The young mother has no conception of the danger which an inflammation of the eyes means to her baby. She has probably never heard that such a condition can threaten the infant's eyes. It is but one of the many new problems which maternity has for her, and only when the truth is told to her that the child, in whom all her hopes had been centered, is hopelessly, irrecoverably blind, does she begin to realise the extent of this frightful affliction. If she chance to learn, as she may, that this calamity was a needless one and might have been avoided by simple precautionary measures which were not taken, to her anguish is added indignation and censure of the physician by whom she considers her trust to have been betrayed.

The third agent concerned is the commonwealth. The loss of sight on the part of an infant makes the individual a state care in some measure for life. For the education of its blind children annually New York alone pays per capita at least three hundred and fifty dollars and a yearly gross sum amounting to much more than one hundred thousand dollars. If, as sometimes happens, the blind citizen is a dependent throughout a long life, the cost of maintainence is not less than ten thousand dollars, and the mere cost in money will be multiplied many times in that a productive factor, in reason of blindness, has been removed from the community.

If, therefore, as an economic proposition, it were realised how vitally it concerns the state that not one child shall needlessly become blind, thereby increasing the public financial burden, there is no doubt that early and effective measures would be instituted to protect the state from this unnecessary and extravagant expenditure of public funds.

It would seem that there are but two reasons why a generally recognised and effective measure for the prevention of a widespread and common cause of blindness is not invariably employed: first, because the dangers of the disease and the value of prevention are not universally known, and, second, because a safe, sterile, simple and free preparation in which the profession and the public have absolute confidence is not always at hand when needed. Concerning the first, various sporadic efforts have been made to inform midwives, who in large cities preside over half at least of the births, of the dangers of sore eyes in the new-born, and eleven states have passed legislative enactments requiring that the midwife shall report each case to the proper health authority and affixing a penalty for the failure to do so. As has been intimated, however, it is not by any means always under the ministration of midwives that these cases occur, and, like all laws behind which is not a strong and well-informed public sentiment, this law is rarely enforced. A more effective method must be devised. Every physician having to do with the parturient woman, every obstetrician, every midwife, must be frequently and constantly advised of the dangers and possibilities of this disease, the necessity of prevention and the value of early and correct treatment. They must have placed in their hands ready for instant use a safe and efficient preparation issued by the health authorities as a guarantee as to its quality and efficiency.

An important step was taken in this direction when a resolution was passed by the House of Delegates, at the annual meeting of the New York State Medical Society, requesting the various health officers of the state to include ophthalmia neonatorum among contagious diseases which must be reported to the local boards of health.

This is, indeed, only a beginning; not only should every case be reported, but the conditions of each eye should be described in the report and accurate records made as to the result. If, then, the sight in one or both eyes is lost, inquiry as to the reason should be instituted. The assurance that such an investigation will certainly follow will inevitably cause a degree of care to be exercised that will immediately lessen the number of cases of blindness due to this cause.

The second essential in order that the cause of infantile ophthalmia be abolished is that a solution of the necessary silver salt be prepared under the authority of some body capable of inspiring universal confidence and that it be distributed by the health department of every state gratuitously to every obstetrician, physician or midwife qualified to care for the parturient woman. The nature of the solution, together with the character of the descriptive card which should accompany it, should be determined by a committee chosen by the President of the American Medical Association and should have among its members at least one representative ophthalmologist, one obstetrician, and one sanitarian. The conclusions of this committee should be reported back to the House of Delegates so that the preparation and its text should carry with it, on the great authority of this association, the assurance that the solution is entirely safe and necessary and that its use should invariably be part of the toilet of every newborn child. The solution, probably silver nitrate, could be put up either by the state itself or by some trustworthy pharmacist at an insignificant cost; its purity and sterility should be vouched for by the board of health of the state. It should be enclosed in specially prepared receptacles, each containing a special quantity and so arranged that it may be used drop by drop. These, properly enclosed accompanied by a brief lucid explanation of the danger of the disease, the necessity of this germicide, the method of its employment, and the right subsequent care of the eyes should be sent to the obstetrician on the receipt of each birth certificate. As with antitoxin, these preventive packages should be placed at various stations where they could be easily obtained, and those by whom they might be used should be urged to secure them. In order that none who should use them should fail to get them, they should be supplied free of cost. Such further supplies as might be needed for further treatment in the proportionally few cases in which prevention did not prove wholly effective should be made readily obtainable at minimum cost. In other words, every facility should be afforded for the early destruction of the infectious germs.

Similar cards should be posted in every maternity hospital and ophthalmic dispensary, and efforts should be made to have the Credé method of prevention by the use of silver nitrate regularly employed as a routine measure in every public and private institution in which children are born.

Special cards should be sent to midwives, giving them more detailed instructions in several languages. These cards should be in the form of return postals, having space for the date on which the ophthalmia appeared, the condition of the cornea, and whether or not preventive measures were employed.

The distribution of these cards should lie with the public health authorities, and the failure to report promptly should constitute a misdemeanor.

FIG. 1—Sample light-proof hermetically sealed ampoules for the distribution of the silver; made in different sizes. The glass tips are to be broken off, the rubber tubing being retained to prevent injury to the eye by untrained or careless hands.

On the filing of each birth certificate the department of health should at once send to the accoucheur an ophthalmia card, with a supply of silver nitrate for immediate use. It would probably, by reason of delay on the part of physician or midwife, be delivered too late for that particular case. Each card, sent, however, would be a constant reminder and the preparation would be on hand to be employed when the next case occurred.

Correspondence with some of the principal pharmacists has shown that the nitrate, which is the most efficient of the silver salts, is also the most permanent. It can be prepared in light-proof ampoules so arranged that a sterile preparation may be easily and safely employed even by inexperienced hands. Such a filled receptacle can be prepared and placed in the hands of the health officers and distributed at a nominal cost. If, however, the sum required to put this valuable preventive in the hands of every accoucheur was much greater than it is it could still be done with great economy to the state.

In the year 1901-2 there were 129 pupils in the New York State School for the Blind; of these the ophthalmic examiner reported 43 as having lost their sight through suppurative ophthalmia. The next year, among 29 new pupils, 11 were catalogued as blind from this cause. In 1903-4, among 24 new pupils, 6 came in the same list, and in 1904-5, of the 23 new pupils, 7 were also so described. A careful re-examination developed the fact that, while all those cases were due to suppurative infections, and, therefore, almost if not all preventable or curable, some of them occurred later than in infancy, but several ophthalmologists agreed that it was quite within the facts to say that 25 per cent. of the pupils in the school had lost their sight as a result of ophthalmia neonatorum. If a like proportion exists in the school in New York City, as is quite probable, these, together with the large number receiving state and city aid through other channels, would easily make an annually increasing budget of now not less than twenty-five thousand dollars paid for the education and maintenance of blind people who, had a tithe of that money been expended for prevention, need never have been blind.

In the city of Buffalo in 1905 there occurred about nine thousand births. During the year four children from the same place were newly entered in the State School for the Blind. Of these one boy had lost his sight through ophthalmia neonatorum. The cost of the maintenance of that one child by the state will far exceed the amount which would have been required to protect the eyes of the entire nine thousand.

As we leave the cities the proportion of children blind from this cause entered throughout the state multiplies prodigiously. There can be no question, therefore, as to the economy on the part of the state in instituting general preventive measures. The cost would be infinitesimal, the benefit prodigious, immeasurable. The present time is peculiarly propitious for the successful execution of such a plan.

I have said that responsibility for the indifference that is annually resulting in such frightful disaster lies primarily with the state, the public and the medical profession.

The state is already aroused to the necessity of taking effective measures to wipe out this controllable plague. Bills have been introduced in the legislature of Massachusetts and of New York providing for the appointment of commissions for the blind, one of whose duties will be to study the causes of unnecessary blindness and to suggest preventive measures.

The public has been awakened and a society for the Improvement of the Condition of the Blind and the Prevention of Blindness has been organised in New York under the distinguished direction of Dr. Lyman Abbott and having on its directorate the names of many eminent citizens. The more generally to popularise its work, a meeting, at which Mark Twain will preside and the Hon. Joseph H. Choate be the chief speaker, will be held at the Waldorf-Astoria, New York, in the present month.

To make these efforts more effective, the hearty cooperation of the medical profession is essential. The magnificent organisation of the American Medical Association makes possible as never before an effective movement to abolish ophthalmia neonatorum as a cause of blindness. Let registration of every case be first secured through the health boards of every state in the Union, then through these same boards have placed gratuitously in the hands of every accoucheur the simple remedy through which protection can be secured, and multitudes whose lives through needless blindness would otherwise result in hopeless failure and untold misery may be saved to their families and the state through the combined efforts of the state, the people and the medical profession. This great thing can be done quickly and effectively. The state and the people are ready. The third, the most powerful element, is the medical profession. Such a happy combination of conditions may never again recur. May not the powerful influence of this Association at this opportune moment be invoked?

454 Franklin Street.

## Figures and Tables

**Fig. 1. f1:**